# TALE-Like Effectors Are an Ancestral Feature of the *Ralstonia solanacearum* Species Complex and Converge in DNA Targeting Specificity

**DOI:** 10.3389/fpls.2016.01225

**Published:** 2016-08-17

**Authors:** Niklas Schandry, Orlando de Lange, Philippe Prior, Thomas Lahaye

**Affiliations:** ^1^Center for Plant Molecular Biology, University of TübingenTübingen, Germany; ^2^UMR Peuplements Végétaux et Bioagresseurs en Milieu Tropical, Centre de Coopération Internationale en Recherche Agronomique pour le Développement – Institut National de la Recherche AgronomiqueSaint-Pierre, France

**Keywords:** crop pathogen, effector adaptation, molecular host–pathogen co-evolution, *Ralstonia solanacearum*, repetitive sequence, transcription activator like effector (TALE), RipTAL, bacterial wilt

## Abstract

*Ralstonia solanacearum*, a species complex of bacterial plant pathogens divided into four monophyletic phylotypes, causes plant diseases in tropical climates around the world. Some strains exhibit a broad host range on solanaceous hosts, while others are highly host-specific as for example some banana-pathogenic strains. Previous studies showed that transcription activator-like (TAL) effectors from *Ralstonia*, termed RipTALs, are capable of activating reporter genes *in planta*, if these are preceded by a matching effector binding element (EBE). RipTALs target DNA via their central repeat domain (CRD), where one repeat pairs with one DNA-base of the given EBE. The repeat variable diresidue dictates base repeat specificity in a predictable fashion, known as the TALE code. In this work, we analyze RipTALs across all phylotypes of the *Ralstonia solanacearum* species complex. We find that RipTALs are prevalent in phylotypes I and IV but absent from most phylotype III and II strains (10/12, 8/14, 1/24, and 1/5 strains contained a RipTAL, respectively). RipTALs originating from strains of the same phylotype show high levels of sequence similarity (>98%) in the N-terminal and C-terminal regions, while RipTALs isolated from different phylotypes show 47–91% sequence similarity in those regions, giving rise to four RipTAL classes. We show that, despite sequence divergence, the base preference for guanine, mediated by the N-terminal region, is conserved across RipTALs of all classes. Using the number and order of repeats found in the CRD, we functionally sub-classify RipTALs, introduce a new simple nomenclature, and predict matching EBEs for all seven distinct RipTALs identified. We experimentally study RipTAL EBEs and uncover that some RipTALs are able to target the EBEs of other RipTALs, referred to as cross-reactivity. In particular, RipTALs from strains with a broad host range on solanaceous hosts cross-react on each other’s EBEs. Investigation of sequence divergence between RipTAL repeats allows for a reconstruction of repeat array biogenesis, for example through slipped strand mispairing or gene conversion. Using these studies we show how RipTALs of broad host range strains evolved convergently toward a shared target sequence. Finally, we discuss the differences between TALE-likes of plant pathogens in the context of disease ecology.

## Introduction

RipTALs from the bacterial plant pathogenic *Ralstonia solanacearum* species complex (Rssc) are homologs of the transcription activator-like effectors (TALEs) from the bacterial plant pathogen *Xanthomonas* (Boch and Bonas, 2010; de Lange et al., 2013). RipTALs, TALEs, *Burkholderia* Bat proteins and the MOrTL proteins from marine microorganisms all bind double-stranded DNA in a sequence-specific, predictable fashion and are collectively referred to as TALE-likes (de Lange et al., 2015). The central repeat domain (CRD) of TALE-likes confers DNA binding and is composed of a variable number of imperfect repeats arranged in tandem, with each repeat 33–35 amino acids in length. Repeat polymorphisms are mostly restricted to positions 12 and 13 in TALE repeats and these positions have thus been termed the repeat variable di-residue (RVD; Moscou and Bogdanove, 2009). In other TALE-likes variation between individual repeats is not restricted to the RVD residues, but nevertheless base preferences can be determined by the RVD-based TALE code (Deng et al., 2012; de Lange et al., 2013, 2014a,b, 2015; Mak et al., 2013). In addition to the RVD-defined base-preference of TALE-likes, the N-terminal regions (NTRs) of TALEs include sequence-degenerate repeat units known to exert a fixed base preference for thymine (T_0_), while the homologous region in RipTALs specifies a preference for guanine (G_0_) (Gao et al., 2012; de Lange et al., 2013).

RipTALs and TALEs are injected into host cells and are able to activate host genes that contain a matching effector binding element (EBE) in their promoter (Mukaihara et al., 2004; Mukaihara and Tamura, 2009; Boch and Bonas, 2010; de Lange et al., 2013). Gene activation is mediated by a transcriptional activation domain located in the C-terminal region (CTR; Van den Ackerveken et al., 1996; de Lange et al., 2013). To date, no RipTAL host target genes have been identified, but the analysis of TALEs has uncovered several examples of host genes that promote disease upon TALE-mediated activation and that have been designated as susceptibility (*S*) genes (Yang et al., 2006; Boch et al., 2014). As a consequence of plant–pathogen co-evolution some lineages within an otherwise susceptible plant species have evolved resistance (*R*) genes that consist of an EBE, embedded in a tightly regulated promoter, and a downstream-encoded executor R protein that triggers a defense reaction when expressed (Gu et al., 2005; Römer et al., 2007; Strauss et al., 2012; Tian et al., 2014; Wang et al., 2015; Zhang et al., 2015). The high similarity between the DNA segments encoding TALE repeats is assumed to provide the basis for rapid evolution of the CRD (Yang and Gabriel, 1995; Yang et al., 2005). Yet, the molecular basis of these changes are not well understood till now. The similarity between repeats is less pronounced in RipTALs than in TALEs (de Lange et al., 2013), suggesting that their evolutionary constraints are different.

Phylogenetically strains of the *R. solanacearum* species complex are classified into four phylotypes (I–IV) that are further sub-divided into sequevars. Assignment of a given strain to a phylotype or sequevar is based on the nucleotide sequence of a set of genomic Rssc reference loci (Fegan and Prior, 2005; Genin and Denny, 2012). Notably the four Rssc phylotypes have geographically separated origins. Phylotype I originated from Asia, phylotype II from the southern Americas, phylotype III is endemic on the African continent, and phylotype IV is found primarily in Indonesia and Oceania (Wicker et al., 2012). Recently, the *R. solanacearum* species complex has been divided into three separate taxonomic species (Safni et al., 2014; Prior et al., 2016). Phylotype II corresponds to the taxonomic species *R. solanacearum*. Phylotypes I and III, that exhibit a broad host range on solanaceous hosts, were assigned to the taxonomic species *R. pseudosolanacearum* and phylotype IV has been assigned the taxonomic species *R. syzygii*, divided into three subspecies (Safni et al., 2014; Prior et al., 2016).

Members of Rssc cause various plant diseases, but all involve an invasion of the vasculature and result in host death. The most prominent is bacterial wilt of solanaceous plants, caused by broad host-range strains. Other economically relevant Rssc-caused diseases include Moko (Southern America) and blood disease (Indonesia) of banana, caused by phylotype II and phylotype IV Rssc strains that have independently undergone host specialization (Remenant et al., 2011; Genin and Denny, 2012; Ailloud et al., 2015).

Effectors are generally important determinants of pathogen host-range and collectively the Rssc possesses an unusually large effector repertoire (pan-effectome; Peeters et al., 2013). However, the number of effectors present in every strain (core-effectome) is much smaller (Peeters et al., 2013). For example, the first sequenced phylotype I strain GMI1000 carries a total of 71 effectors (Peeters et al., 2013)

Studies on the diversity and function of RipTALs from Rssc were focused on phylotype I (Heuer et al., 2007; de Lange et al., 2013; Li et al., 2013), and thus little is known on RipTALs from other phylotypes. Previous work has shown type III secretion system-dependent translocation of phylotype I RipTALs and revealed that a *ripTAL* knockout in Rssc strain GMI1000 leads to reduced competitive fitness of the mutant strain *in planta* (Mukaihara et al., 2004; Mukaihara and Tamura, 2009; Macho et al., 2010).

In this work, we dissected the phylogenetic and functional diversity of RipTALs across the whole Rssc. We predict and experimentally study RipTAL EBEs and uncover that some RipTALs are able to target the EBEs of other RipTALs, a phenomenon that we refer to as cross-reactivity. Notably RipTALs within a given cross-reactivity group typically originate from strains with the same host specialization, suggesting conserved RipTAL host targets within these strain groups. Finally, inspection of *ripTAL CRDs* uncovers unique, thus far not recognized patterns in their sequence composition. Those patterns facilitate the identification of mechanisms, such as slipped-strand mispairing and segmental gene conversion, shaping the *ripTAL CRD*, uncovering major differences between *ripTAL* and *TALE CRD* regarding their evolution. Our insights provide the basis for a better understanding of the evolutionary constraints shaping TALE-likes and should enable us to anticipate changes in these effectors and thus foster design of durable synthetic *R* genes mediating recognition of TALE-likes.

## Materials and Methods

### Strain Selection

We acquired genomic DNA from strains covering all four phylotypes of the Rssc and representing a broad geographic distribution (Supplementary Table S1). The rationale behind strain selection differed depending on the phylotype.

#### Phylotype II

This taxonomic species is large and well studied compared to other phylotypes, but based on available genome sequences *ripTALs* are apparently restricted to banana infecting strains in this phylotype. These include strain Molk2 (Remenant et al., 2010) from Indonesia, for which the *ripTAL* has been previously described (Li et al., 2013) as well as Grenada91 with a partially assembled *ripTAL* (Ailloud et al., 2015). In order to confirm these findings for phylotype II we studied a set of five strains, including three for which genome sequence is available, among them Molk2, known to contain a *ripTAL* (Li et al., 2013), and two others (K60, UW551) previously shown to not contain *ripTALs*.

#### Phylotypes I and III

We had previously studied RipTALs of phylotype I strains from China (de Lange et al., 2013) and focused our attention for this study on phylotype I strains from Mayotte Island in the Indian Ocean. This population has a broad host range, infecting diverse solanaceous crops, and is well characterized. The strains included in our analysis span all Mayotte sequevars [subclades within phylotypes (Genin and Denny, 2012)].

Phylotype III strains occur predominantly in Africa and, in contrast to the other phylotypes, are poorly studied. No *ripTAL*s have been reported for phylotype III strains. We therefore placed particular emphasis on this phylotype, screening 23 strains from seven countries, nine hosts and at least nine sequevars (some strains await sequevar assignment, Supplementary Table S1).

#### Phylotype IV

Phylotype IV is predominately found in Indonesia and Oceania. Some strains of this phylotype display an unusual degree of host-specificity compared to other Rssc strains (Remenant et al., 2011; Ailloud et al., 2015). These include banana-infecting Blood Disease Bacterium [BDB, also classified as *Ralstonia syzygii* subsp. *celebesensis* (Safni et al., 2014)], and clove-infecting strains [classified as *R. syzygii* subsp. *syzygii* (Remenant et al., 2011; Safni et al., 2014)]. BDB strain R229 and tomato infecting strain Psi07 (RUN83) have previously been indicated to contain full-length *ripTALs* based on genome sequences. We analyzed 14 phylotype IV strains, with an emphasis on host-specialized strains of the subspecies *syzygii* and *celebesensis.*

### PCR Screening of gDNAs for, and Cloning of ripTALs into *In planta* Expression Vectors

Genomic DNA of Rssc strains was extracted using Wizards^®^ gDNA extraction kit (Promega). Primers allPT-F and -R were designed based on ripTAL sequences found in public genomes (sequences available^1^ Peeters et al., 2013). PCRs to screen for *ripTAL*s were performed with primers allPT-F and allPT–R (Supplementary Table S2), using Phusion polymerase (NEB) in GC buffer (NEB), supplemented with 20% preCESI (Ralser et al., 2006) and PCR clean up (Fermentas) was performed if a fragment >1.5 kb was visible on an Agarose gel. PCR products were sequenced using primers allPTRepF and R (Supplementary Table S2). Sequencing produced clear peaks, indicating that a single gene was amplified. Repeats were annotated to identify the RVD composition. The sequence of *ripTALIII-1* was elucidated by genome walking (Leoni et al., 2011) from the *CRD* toward *NTR* (5′) and *CTR* (3′).

RipTAL genes with RVD compositions not previously described, were amplified from genomic DNA with class specific primers (Supplementary Table S2), generating BsaI flanked fragments while removing internal BsaI recognition sites after cut-ligation into pENTR-CACC-GW-AAGG (de Lange et al., 2014b). Cloned RipTALs were validated by sanger sequencing and transferred into pGWB641 (Nakamura et al., 2010) via an LR Gateway cloning reaction (Life Technologies).

*ripTAL* sequences have been deposited at ENA and are accessible with accessions LN874044-63.

### Prediction and Cloning of Effector Binding Elements into the *Bs3* Promoter

Effector binding elements were predicted using the RipTAL code (de Lange et al., 2013), which closely matches the TALE code. In the case of RipTAL repeats with previously uncharacterized RVDs, data from TALE DNA binding domains was used (Boch et al., 2009; Moscou and Bogdanove, 2009; Miller et al., 2015). EBEs were cloned as described previously (de Lange et al., 2013) and subsequently transferred into pENTR-ccdB-uidA via cut-ligation.

### Protoplast Transfection

*Arabidopsis thaliana* root cell culture was maintained as described (Li et al., 2010). Protoplasts were pelleted by centrifugation at 50 × *g* for 5 min and resuspended in MM (0,4 M Mannitol, 5 mM MES, pH 6) at a cell density of 10^7^ cells per ml. Thirty microliters of protoplasts were transferred into wells of a 2 ml deep well plate together with 3 μg of RipTAL expression plasmid, 3 μg of GUS-reporter plasmid and 1 μg of luciferase expression plasmid. Thirty microliters of PEG1500 were added to each well and mixed gently. After incubation for 5 min 30 μl of MM were to each well added to stop the transformation. Then 300 μl of K3 (Schütze et al., 2009) were added to each well and the plate was sealed with parafilm and stored at 21°C in the dark.

Twenty hours after transfection 1.7 ml of MMg (MM with 15 mM MgCl_2_) were added to each well and cells were pelleted at 400 × *g* for 10 min at room temperature. Supernatant was removed to leave 70 μl volume. Protoplasts were lysed in 70 μl of 2x Cell culture lysis reagent (Promega, plus 1 Roche EDTA-free protease inhibitor tablet per 20 ml) on ice by pipetting up and down 10 times per well, whilst avoiding the introduction of air bubbles. One hundred microliters of lysed protoplasts were transferred into a PCR plate and stored on ice for 30 min. After centrifugation at 4000 × *g* for 30 min at 4°C the supernatant was transferred into a new plate and used to determine Luciferase and GUS activity in a plate reader (Berthold). GUS enzyme activity was measured as 4-MU fluorescence (excitation at 355 nm, emission at 460 nm) at 37°C over 80 min for 10 μl of protoplast supernatant in 90 μl GUS buffer (100 mM Tris-HCl, 2 mM MgCl_2_, 4 mM 4MUG at pH 8.2). A single reading of Luciferase activity was carried out with 50 μl of reconstituted Promega luciferase assay reagent injected into 10 μl of protoplast supernatant.

### Data Analysis of Protoplast GUS Assays

Data analysis was performed using Excel (Microsoft) and R^2^ and figures were created with additional R packages RColorBrewer, gplots, and lattice. The change in 4-MU fluorescence intensities over a 20-min interval were adjusted to a per minute value. These values were then normalized for transfection efficiency by dividing by the corresponding luciferase activity.

For the background measurements using AvrBs3 and respective EBEs, five replicates were performed and averaged (see exclusion criteria in the next sentence), and this average was used to determine the fold change of all other measurements on that same EBE done on the same day and the same plate. Individual wells, which had a luciferase reading below 1000 (fluorescence units), were excluded from the analysis as this indicates poor transfection efficiency. Experiments, consisting of five replicates, were repeated at least twice and all results are shown.

Statistical analyses were done using R. To assess normality of GUS data, density distributions were plotted and visually examined. Distributions appeared to be non-normal and asymmetric (skewed), and therefore a non-parametric statistical test (Wilcoxon rank sum test, as implemented in R package stats with function wilcox.test) was used, and median values are used as a measure of center of distributions (Krzywinski and Altman, 2014).

### Confocal Laser Scanning Microscopy (CLSM)

Microscopy of *A. thaliana* root-cell culture protoplasts was carried out 24 to 36 h after transfection with pGWB641 *ripTAL* constructs, which results in expression of C-terminal YFP fusion proteins. To mark out nuclei within the cell, plasmid pCF205 bearing a 35-S driven mCherry-NLS construct (Llorca et al., 2015) was co-transfected together with each *ripTAL* expression plasmid. A Leica DMI6000B-CS SP8 confocal laser scanning microscope with an HC PL APO CS2 40x/1.10 water objective was used for imaging. Excitation was performed at 513 and 594 nm using Argon and HeNe Lasers for YFP and mCherry, respectively. Emission spectra were 522–556 nm for YFP and 604–627 nm for mCherry. A single focal plane was used to prepare images shown. Image analysis and processing of imaging stacks was performed using Fiji (Schindelin et al., 2012).

### Bioinformatic Analysis

Sequence analyses, including ClustalW alignments were performed using CLC Main Workbench v 7.6.1 (Qiagen, Aarhust). Individual *repeat* sequences were extracted from repeat arrays using R with the Biostrings package. Nucleotide sequences used for the per-repeat comparisons, and calculations of GC content were those of *ripTALI-1_*RUN2108*_, ripTALI-8_*RUN2127*_, ripTALI-9_*RUN64*_, ripTALII-1_*Molk2*_, ripTALIII-1_*RUN369*_, ripTALIV-1_*RUN83*_*, and *ripTALIV-2_*RUN1348*_*.

## Results

### RipTALs Are Found in all Rssc Phylotypes

We studied a collection of 54 strains, spanning all Rssc phylotypes (Supplementary Table S1). A particular emphasis was given to phylotype III, as there was no RipTAL previously discovered in this phylotype. Further details on the rationale behind strain selection are given in the “Materials and Methods” section.

**FIGURE 1:**
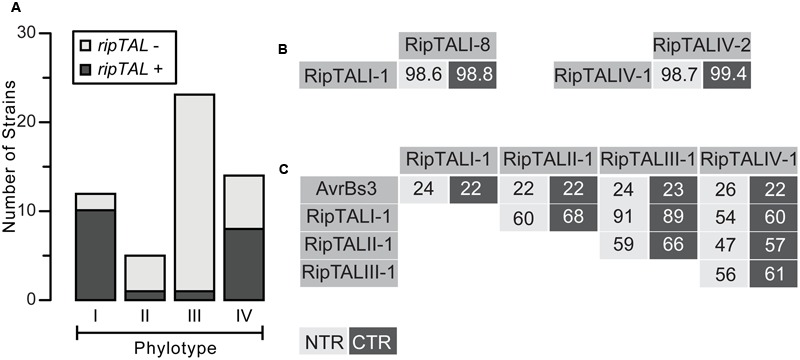
**RipTAL abundance differs across *Ralstonia solanacearum* phylotypes but RipTALs sequences are similar within and different across phylotypes. (A)** Abundance of *ripTAL*s in strains from distinct phylotypes. The assessment is based on PCR analysis with primers flanking the *repeats* and was carried out on a broad collection of Rssc strains covering all phylotypes and different degrees of host adaptation. **(B,C)** Pairwise NTR and CTR sequence identities of depicted RipTALs from closely related Rssc strains **(B)** and RipTALs from different Rssc phylotypes or the *Xanthomonas* TALE AvrBs3 **(C)** are given in percent. Light and dark gray background indicates identities in the NTR and CTR, respectively.

Within this manuscript unambiguous discrimination between protein domains and DNA sequences encoding these protein domains, such as CRDs or repeats, is achieved by the use of italic font for DNA. We first analyzed all strains for presence of a *ripTAL* (**Figure 1A**; Supplementary Table S2). Short 20–30 bp regions flanking the *CRD*, are conserved among *ripTALs* from sequenced genomes and were used to deduce primers. We detected a *CRD* amplicon for most phylotype I strains (10/12) and phylotype IV strains (8/14). Within the five investigated phylotype II strains only Molk2 tested positive for a *ripTAL*. Analysis of 23 phylotype III strains uncovered a *ripTAL* only in strain RUN369. This is, to the best of our knowledge, the first full-length *ripTAL* reported for a phylotype III strain (Supplementary Table S1, Guidot et al., 2007). In summary, our screen uncovered for each Rssc phylotype at least one strain containing a *ripTAL.* Yet *ripTAL* abundance differs significantly across the four phylotypes. This conforms with the differential abundance between phylotypes known for other Rssc effectors (Peeters et al., 2013).

### RipTALs Can Be Divided into Four Classes, in Line with Strain Phylogeny

We next classified RipTALs based on sequence identities. To do this we amplified, cloned, and sequenced full length *CDS*s. We then compared translated amino acid sequences of NTRs and CTRs and found that RipTALs from strains within the same phylotype show very high sequence identities. For example, RipTALs from phylotype I are 98.6% and 98.8% identical for the NTR and CTR, respectively (**Figure 1B**). Cross-phylotype comparisons of RipTAL NTR and CTR sequences uncovered patterns of sequence identities that coincide with Rssc phylogeny. For example, RipTALI-1 and RipTALIII-1 from the closely related phylotypes I and III show identities of 91% (NTR) and 89% (CTR). Accordingly, RipTALs of distantly related phylotype II and IV strains show only 47% (NTR) and 57% (CTR) identity (**Figure 1C**). However, all RipTALs we examined are more similar to one another than any is to the representative *Xanthomonas* TALE AvrBs3 (22–26% identity; **Figure 1C**). Based on this we use phylotype designations (I–IV) to refer to the RipTAL class found in strains of that phylotype.

### DNA Binding Domain Diversity Is Limited

Defined here as the number and order of RVDs in a repeat array, the RVD composition can be used to infer EBEs of TALEs and RipTALs (Boch et al., 2009; Moscou and Bogdanove, 2009; de Lange et al., 2013). We introduce here a new RipTAL designation scheme that integrates information on (i) the sequence composition of N- and C-terminal regions (class designation), (ii) DNA target preference and the (iii) donor strain, in one term. The form suggested is RipTALX-N_Strain_, where X and N are Roman and Arabic numerals denoting RipTAL class (see the section, RipTALs Can Be Divided into Four Classes, in Line with Strain Phylogeny) and RVD composition, respectively, and where the donor strain designation is displayed in subscript font. This proposed change in RipTAL nomenclature is in accordance with a recent proposition on the nomenclature of Rssc effectors (Peeters et al., 2013), but requires that some previously described RipTALs be renamed (Supplementary Figure S1) (de Lange et al., 2013). We believe our new classification provides useful functional information at a glance.

**FIGURE 2:**
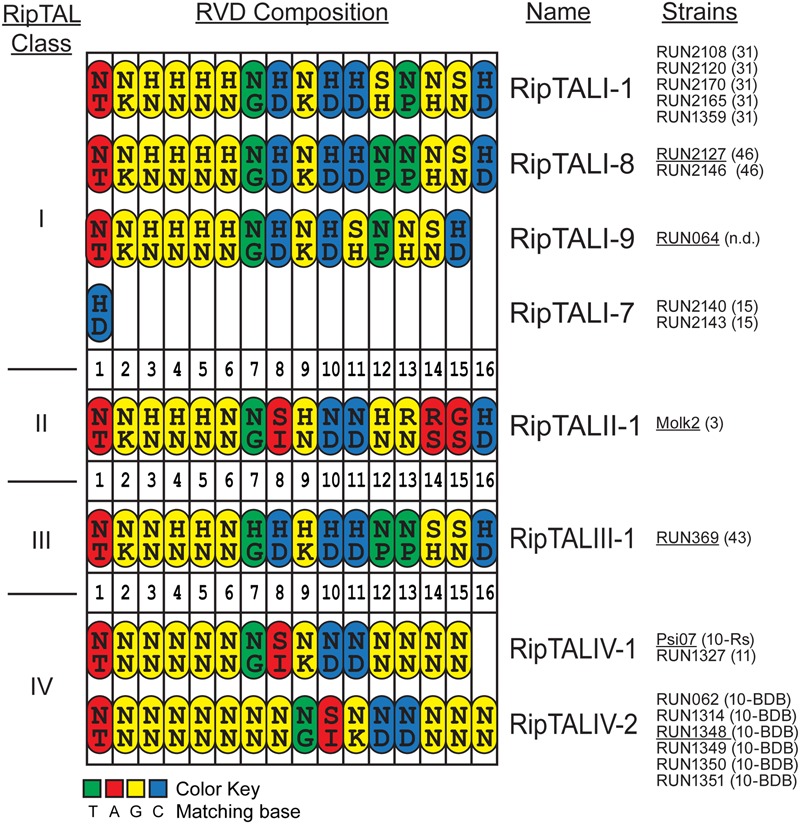
**Comparison of RVD compositions of novel RipTALs across all four *Ralstonia solanacearum* phylotypes reveals limited diversity.** Cartoon displays RVD compositions of newly identified RipTALs separated by class. Each repeat is depicted as an oval. Capital letters inside the repeats indicate amino acids (single letter code) in position 12 and 13 (RVD) of each repeat. Repeats are color-coded based on the preferred base of repeat residue 13, which is the key base specificity determinant, with a color code given at the bottom. Strains bearing a particular *ripTAL* are given next to the RipTAL identifier in black text. Text in brackets gives the sequevar of this strain, n.d. indicates that this strain has not been clearly assigned to a sequevar. Underlined strain name indicates that the given *ripTAL* was studied as a representative in functional assays. RipTALI-2 to RipTALI-6 were described previously (de Lange et al., 2013) and are shown in Supplementary Figure S1.

In total, we found eight distinct RVD compositions in this study: four among the RipTALIs, a single RipTALII, the first RipTALIII and two RipTALIVs. RipTALs with an identical RVD composition are grouped together (**Figure 2**). For example, the RipTALs of phylotype I strains RUN2108 and RUN2120, have the same RVD composition and are therefore classified as RipTALI-1_RUN2108_ and RipTALI-1_RUN2120_ (**Figure 2**), respectively.

Comparison of RipTAL CRD uncovers a remarkably low diversity in their RVD composition not only within but also across RipTAL classes. For example, RipTALI-8 and RipTALIII-1 are near identical with respect to their RVD composition despite the fact that they originate from distinct phylotypes (**Figure 2**).

For subsequent functional assays we cloned one representative full-length CDS of each RVD composition. RipTALI-1_GMI1000_ was previously cloned (previously designated as Brg11; Cunnac et al., 2004; de Lange et al., 2013) and we further cloned RipTALI-8_RUN2127_, RipTALI-9_RUN64_, RipTALII-1_MOLK2_, RipTALIII-1_RUN369_, RipTALIV-1_Psi07_, and RipTALIV-2_RUN1348_. For simplicity, we refer to those RipTALs from hereon without stating their strain designation.

### RipTALs of all Classes Localize to the Nucleus in *Arabidopsis* Protoplasts

**FIGURE 3:**
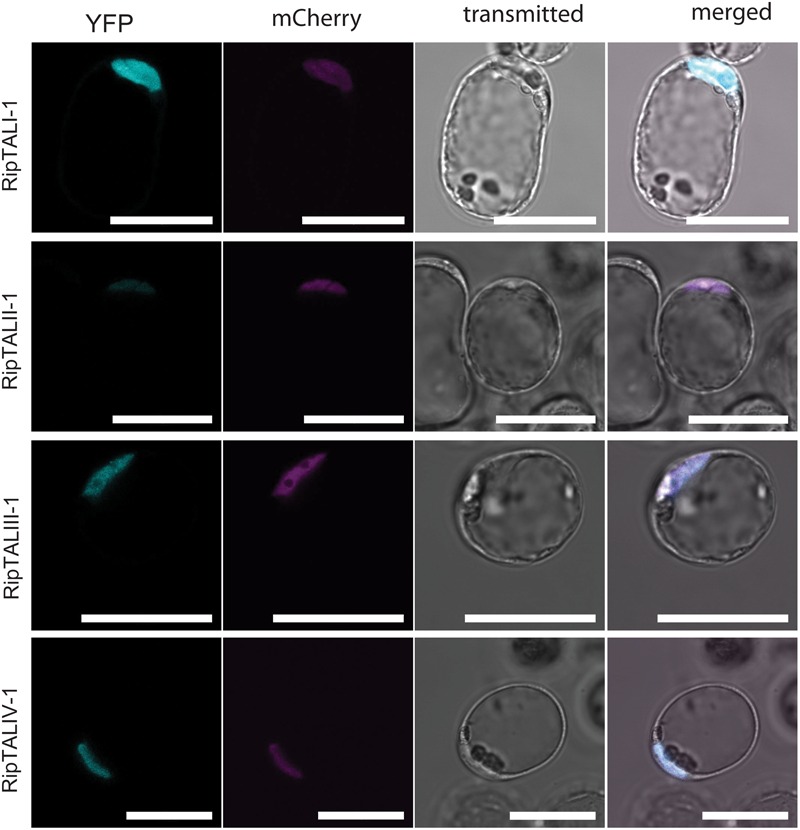
***In planta* expressed RipTALs of all classes show nuclear localization.** Confocal laser scanning microscopy images of *Arabidopsis thaliana* protoplasts expressing depicted YFP-tagged RipTALs and a nuclear-targeted mCherry. Scale bars represent 50 μm.

We carried out a molecular characterization of the newly cloned *ripTAL*s, starting with *in planta* subcellular localization of corresponding RipTALs. To do this, the *ripTAL* CDSs were transferred to a T-DNA vector in between a constitutive cauliflower mosaic *35S* promoter (5′) and *YFP* CDS (3′) for constitutive *in planta* expression of a YFP-tagged RipTAL in each case (Nakamura et al., 2010). Upon transfection of *Arabidopsis thaliana* protoplasts the subcellular localization of RipTAL-YFP fusion proteins was assessed using confocal laser scanning microscopy. A plasmid encoding a nuclear-targeted mCherry was co-transfected to visualize the nucleus in each case (Llorca et al., 2015). We found that all tested RipTAL classes localize exclusively to the nucleus (**Figure 3**; Supplementary Figure S2), in agreement with previous studies on class I RipTALs (de Lange et al., 2013; Li et al., 2013).

### RipTALs Activate Predicted EBEs with a Conserved G_0_ Preference

RipTALI-7 was not included in this and subsequent functional studies, as we previously showed that RipTALs of that RVD composition do not act as transcriptional activators (de Lange et al., 2013).

We predicted the EBEs for all newly identified RipTALs and cloned each EBE into the transcriptionally silent pepper *Bs3* promoter, replacing the EBE of AvrBs3 (Römer et al., 2007), upstream of an *uidA* (GUS) reporter gene. Next, we tested the ability of RipTALs to transcriptionally activate promoters containing corresponding predicted EBEs in *A. thaliana* protoplasts.

**FIGURE 4:**
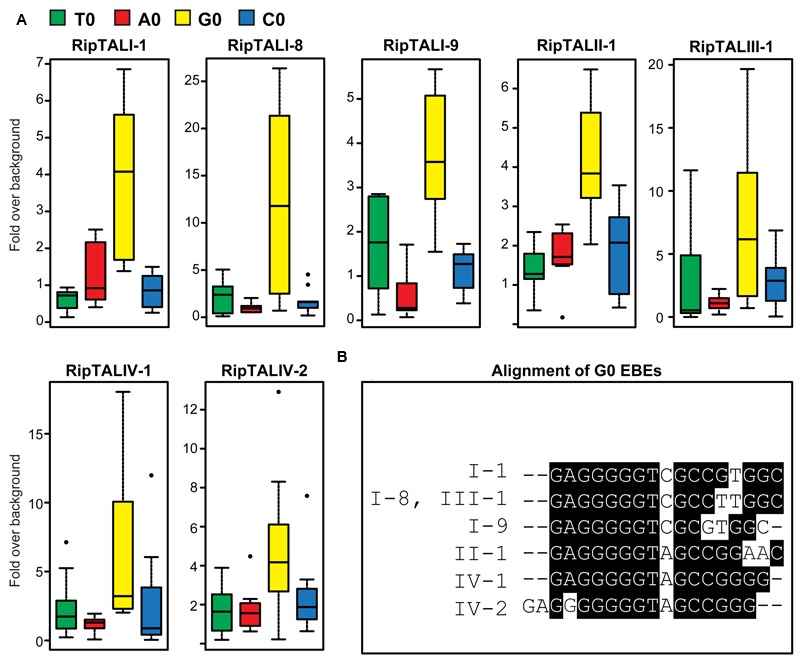
**All RipTALs activated promoters bearing predicted G_0_ Effector Binding Elements (EBEs). (A)** All RipTALs were tested against pepper *Bs3* promoter derivatives, bearing the RipTAL EBE in place of the AvrBs3 binding site, preceded by the given base (indicated by color-coded boxplots) upstream of a *uidA CDS*. Background levels were determined using the same promoter-reporter in combination with AvrBs3. Experiments were repeated twice and all results are shown. G_0_EBEs were activated significantly (*p* < 0.01, Wilcoxon rank-sum test), while the others were not. **(B)** Boxshade alignment of G_0_EBEs corresponding to depicted RipTALs.

Previous work on class I RipTALs had shown that the RVD-defined EBEs mediate activation only if preceded by a guanine base (base 0; de Lange et al., 2013). The base 0 preference in class I RipTALs is mediated by a domain within the NTR (de Lange et al., 2013). Our sequence analysis revealed polymorphisms between RipTAL classes in the NTR (**Figure 1C**). To test if these NTR polymorphisms would affect base 0 preferences, we constructed EBEs not only with a G_0_, but also with A_0_, C_0_, and T_0_ variants to interrogate the base 0 preferences. GUS measurements of the RipTAL-promoter combinations showed in every case that the tested RipTAL was able to activate a promoter containing its predicted EBE (**Figure 4A**). Moreover, all RipTALs tested activated their G_0_EBEs most strongly (**Figure 4A**). Of the EBEs preceded by a base other guanine, none was activated significantly (*p* < 0.01 determined by Wilcoxon rank-sum test). Accordingly, G_0_EBEs were used for all subsequent assays.

### RipTALs Display Overlap in Their Activation Profiles as defined by Their RVD Composition

**FIGURE 5:**
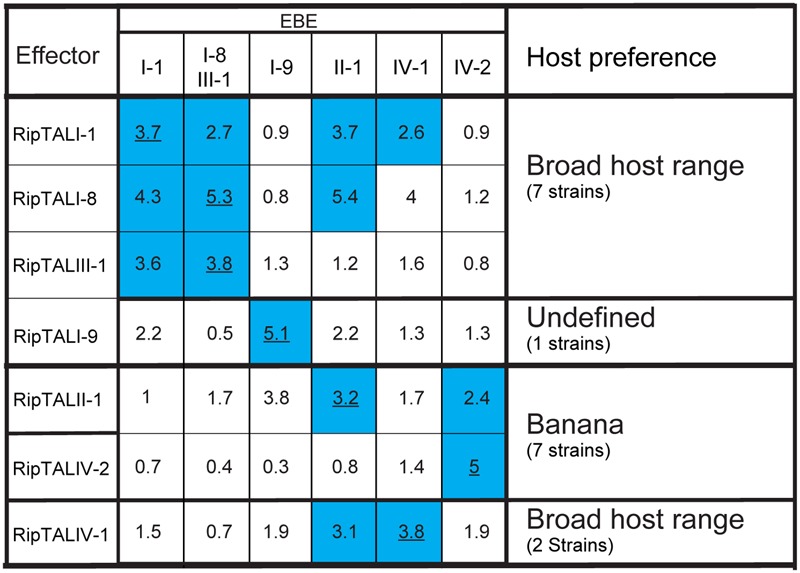
**RipTALs form functional groups based on cross activation.** Increase in GUS-reporter activity for RipTALs on promoters with predicted EBEs. Columns indicate promoter-embedded EBEs tested. The last column provides information on the natural host range of the RipTAL bearing strains identified in this study. All full-length RipTALs were tested against all EBEs. For each RipTAL-EBE combination the median fold activation is given. Underlined values indicate predicted RipTAL-EBE combinations. Blue background is used for RipTAL-EBE combinations that were significantly greater than 1 (*p* < 0.01, determined by Wilcoxon rank-sum test).

We noted that some predicted RipTAL EBEs differ by only single nucleotide polymorphisms (SNPs; **Figure 4B**). We hypothesized that RipTALs would target predicted EBEs differing from their own EBE by only one or a few SNPs. In contrast, RipTAL repeat arrays are not expected to accommodate insertions or deletions in their EBE (Richter et al., 2014). To test this hypothesis, we transfected *Arabidopsis* protoplasts with all possible combinations of RipTALs and reporter constructs containing distinct EBEs, and measured resulting GUS activities (**Figure 5**). As anticipated, we found in many cases that RipTALs activated not only the promoter construct containing its cognate EBE but also those bearing EBEs with one or a few SNPs. Such cross-reactivity was observed for example in RipTALI-1 and RipTALI-8, both originating from broad host-range strains. Both RipTALs were able to activate EBE_I-1 and EBE_I-8 reporters, since the corresponding EBEs differ by one SNP only (**Figure 4B**). By contrast RipTALI-1 and RipTALI-8 are both unable to activate the reporter construct containing EBE_I-9 (**Figure 5**), differing in multiple positions (**Figure 4B**).

Inspection of all RipTAL-EBE combination uncovers two major cross reactivity groups. The first group contains RipTALs I-1, I-8, and III-1 all originating from broad host-range strains activating promoters containing each other’s EBEs.

The second cross activation group unites RipTALs II-1 and IV-2 cloned from banana-specialized phylotype II and phylotype IV strains that both target a common promoter (**Figure 5**). This observation was unexpected given the marked differences in the RVD composition of RipTALII-1 and RipTALIV-2 (**Figure 2**).

### Repeat 8 with the RVD HD Does Not Discriminate between Adenine and Cytosine Bases

**FIGURE 6:**
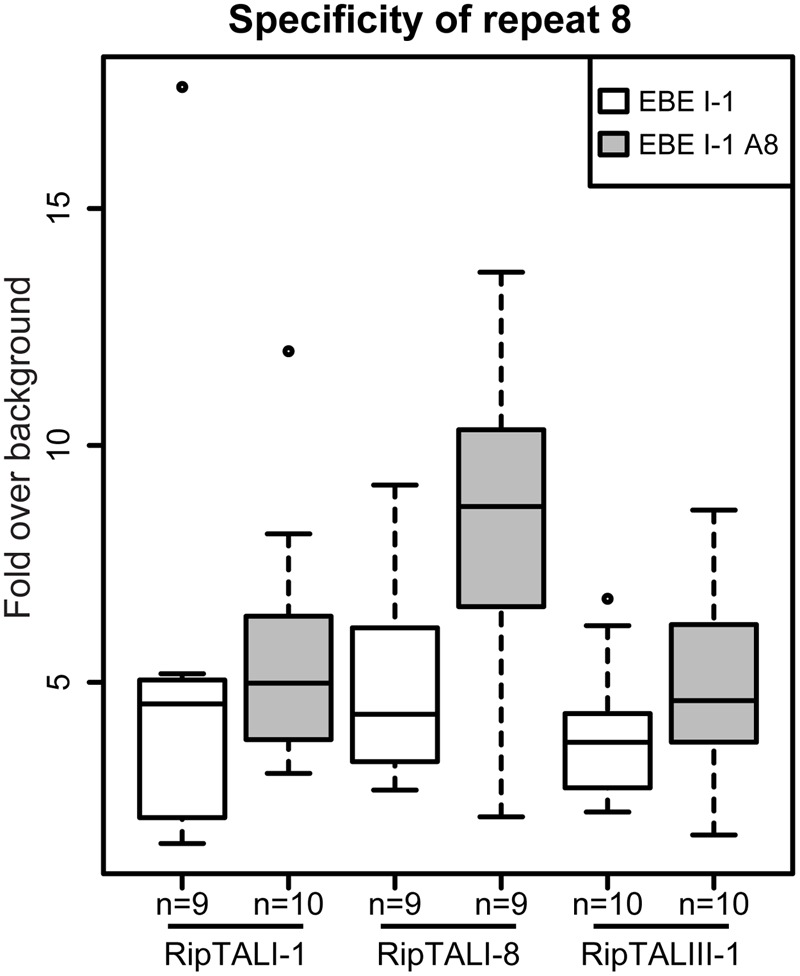
**HD repeat 8 of RipTALs from broad host-range strains is unable to discriminate between adenine and cytosine.** White boxplots show fold GUS change over background on the RipTALI-1 EBE reporter. Gray boxplots show fold GUS change over background for the same EBE where base 8 was changed from cytosine to adenine. The number of replicates is given below each plot.

Our experiments revealed many cases of cross-reactivity of RipTALs. We noted that RipTALI-1, which has a repeat with RVD HD at position 8, was able to activate promoters containing EBE_II-1 and EBE_IV-1 (**Figure 5**) despite both containing an adenine in place of cytosine (**Figure 4B**) at position 8. In previous studies synthetic trimers of this RipTAL HD repeat were tested in the context of an AvrBs3 scaffold and showed a strong preference for a cytosine base trimer (de Lange et al., 2013) suggesting that pairing of this HD repeat to adenine should cause a reduction in promoter activation. To test if this prevalent adenine/cytosine polymorphism in our predicted EBEs actually has any significant impact on the recognition by distinct RipTALs we created an EBE_I-1 derivative where cytosine 8 is replaced by adenine. This modified EBE (EBE_I-1_A8) was tested in combination with RipTALI-1, RipTALI-8, and RipTALIII-1. Analysis of reporter activation levels revealed in all cases that the promoter containing EBE_I-1_A8 was activated to equivalent or higher levels than the promoter containing EBE_I-1 (**Figure 6**). These data suggest that the HD repeats 8 of RipTALs I-1, I-8, and III-1 are compatible with both, cytosine and adenine in their native CRD context, which rationalizes the considerable cross-reactivity observed for these RipTALs (**Figure 5**). Given that the investigated HD repeat 8 was mostly incompatible with adenine in the context of an AvrBs3 scaffold, these data indicate context-dependency of this RipTAL HD repeat. Notably similar context dependency has been observed previously for HD TALE repeats that in a certain context showed preference for adenine instead of cytosine (Meckler et al., 2013; Miller et al., 2015).

### Individual *ripTAL* Repeats from the Same CRD Show Different Degrees of Sequence Identity

**FIGURE 7:**
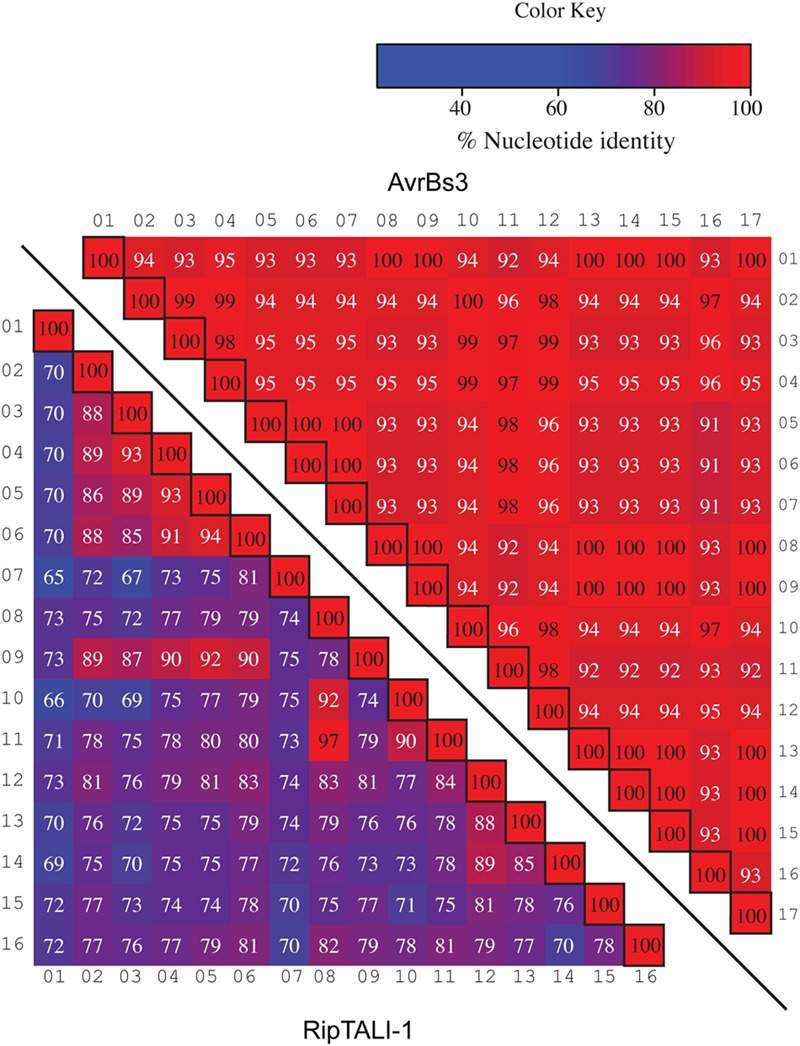
**Comparison of *repeats* within a *TALE* a *ripTAL* uncovers pronounced differences in their inter-*repeat* identities.** Individual *repeats* of the *TALE avrBs3* (upper right) and *ripTALI-1* (lower left) were, aligned, ordered as in their native CRD, and pairwise identities were calculated (percentages in cells). Values <97% are displayed in white font, values ≥97 in black font. Color-coding of cells indicates identities between two given *repeats*, with a color key given upper right. Black-framed cells indicate comparison of a repeat against itself. Numbers above or adjacent to repeats indicate the position of the repeat within the given array.

**FIGURE 8:**
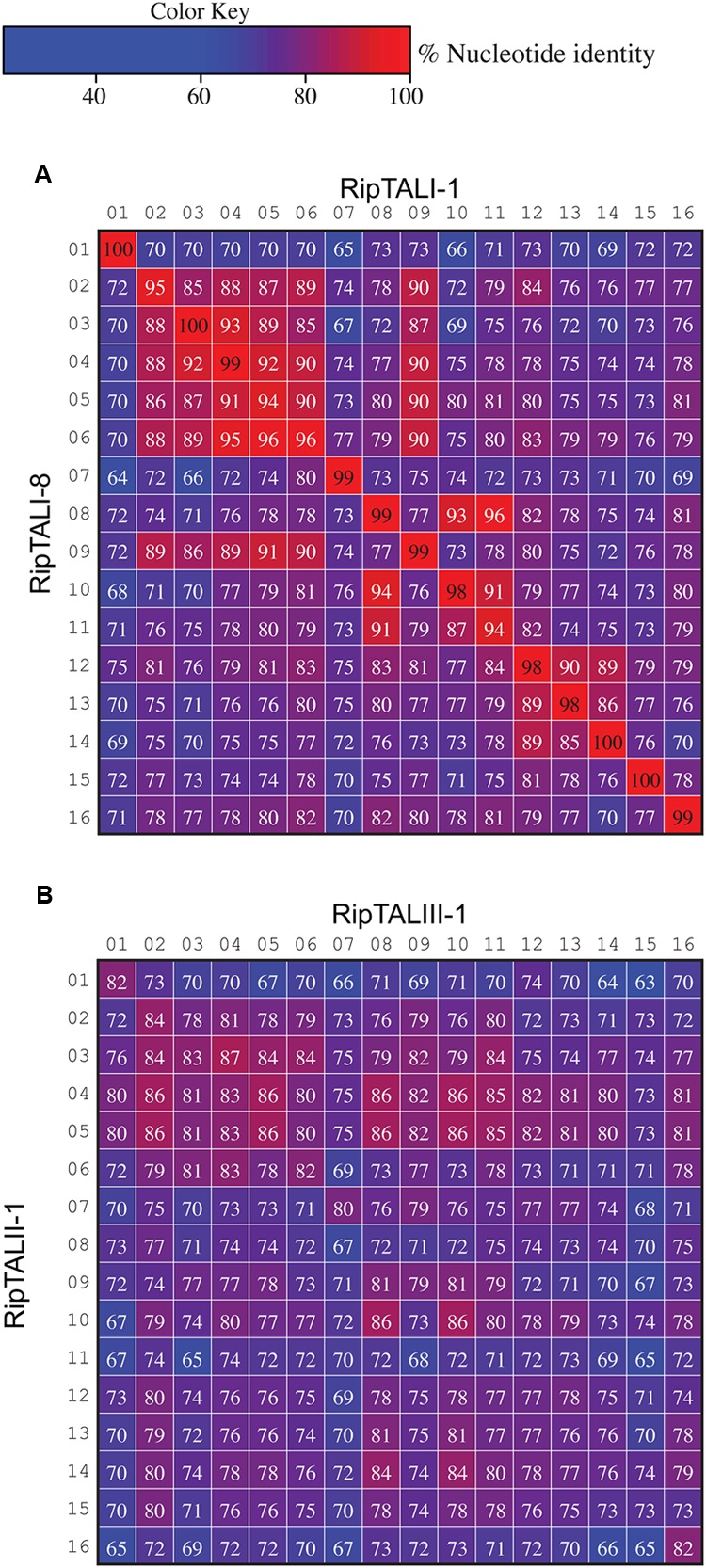
**Inter-*repeat* comparisons of *RipTALs* from closely related Rssc strains uncovers position-dependent conservation (PDC) of *repeats*.** Individual *repeats* were aligned and pairwise identities were calculated. *Repeats* are shown in their native order. Cells are color coded by their percentage identities value according to the color code to the right. Identities <97% are displayed in white font, those ≥97% in black font. Numbers indicate the position of the repeat within the CRD. **(A)** Comparison between all *repeats* of the *CRD* of *ripTALI-1* (columns) versus all *repeats* of the *CRD* of *ripTALI-8* (rows). **(B)** Comparison between all *repeats* of the *CRD* of *ripTALIII-1* (columns) versus all *repeats* of the *CRD* of *ripTALII-1* (rows).

Previous work on *ripTAL*s from phylotype I suggested that *ripTAL CRDs* are subject to recombinatorial mechanisms and are evolving at higher rates relative to the genome (Heuer et al., 2007). Yet, this early study was based on *CRD* length only since the nucleotide composition was not available at that time. We therefore compared sequence composition of individual *repeats* within and across *ripTAL CRD*s to study their evolution. Analysis of all *repeats* of the *TALE* representative *avrBs3* to each other shows pairwise *repeat* identities ranging from 91 to 100% (**Figure 7**) in line with previous analysis of *avrBs3* and other *TALE repeats* (Bonas et al., 1989; Pérez-Quintero et al., 2015). In contrast *ripTAL repeat* identities are far more scattered. For example, in *ripTALI-1* identities range from 65% (repeat 7 vs. repeat 1) up to 97% (repeat 8 vs. repeat 11; **Figure 7**). The pronounced differences in sequence identity between *ripTAL repeats* provide a tool to study their evolutionary relationships. In contrast, near-identical *repeats* of *TALEs* are poorly suited for such evolutionary investigations. We first compared *repeats* of pairs of closely related (based on strain phylogeny) *ripTAL CRDs*: we compared *ripTALI-1* versus *ripTALI-8* (**Figure 8A**), as well as *ripTALIV-1* versus *ripTALIV-2* (Supplementary Figure S4b). In both cases *repeats* occupying the same position in each *CRD*, generally show a high degree of sequence conservation. This is clearly observable as a diagonal line of over 90% sequence identity (indicated in red color) in **Figure 8A** and Supplementary Figure S4b. This implies that *ripTAL repeats* generally retain fixed positions over time. This position-dependent conservation is not observed when *ripTAL repeats* from distantly related strains are compared (**Figure 8B**), perhaps indicative of a large degree of sequence drift between these *ripTAL* genes.

### Different Pathways of Molecular Evolution Have Left Footprints in *ripTAL CRDs*

The evolution of *Xanthomonas* TALEs is difficult to study, since their repeats contain few to no polymorphisms, and therefore phylogenies are challenging to deduce (Pérez-Quintero et al., 2015). Since *ripTAL* repeats exhibit greater sequence diversity than *TALE repeats* (de Lange et al., 2013) we set out to investigate how *ripTAL repeat arrays* evolve. To do this, we inspected *repeats* of closely related *ripTALs* to deduce the molecular mechanisms underlying *ripTAL repeat array* evolution. We found evidence of three distinct molecular mechanisms shaping *ripTAL CRD*s: single nucleotide exchanges, slipped-strand mispairing and segmental gene conversion, presented below.

#### Nucleotide Exchanges in Codons Translating into RVDs

**FIGURE 9:**
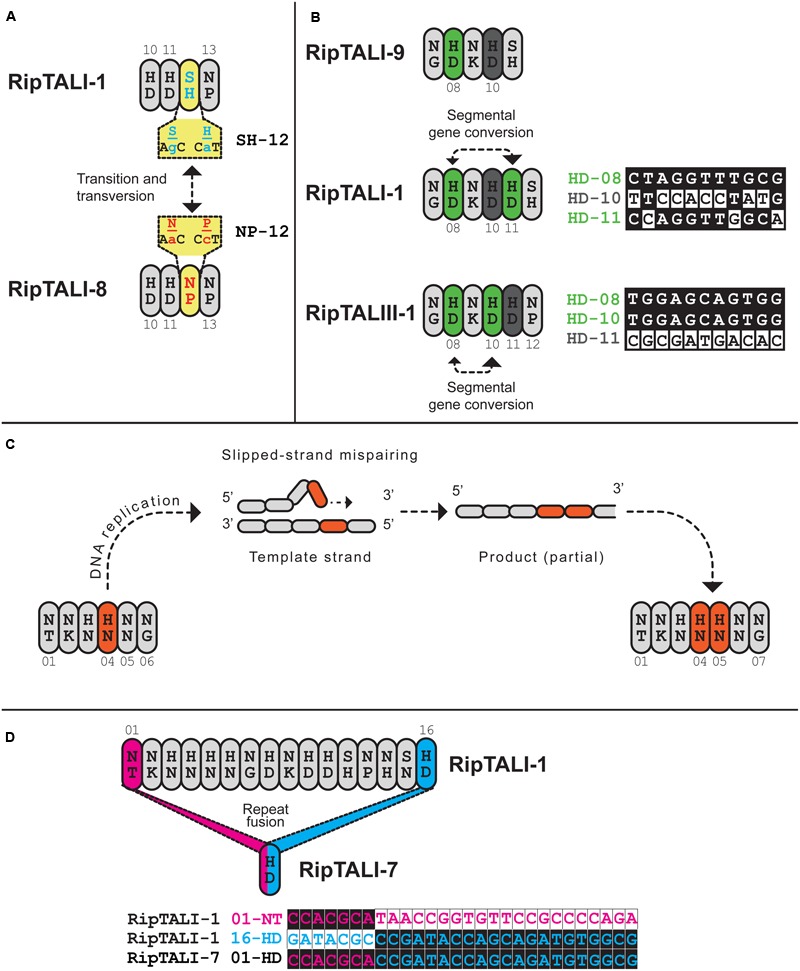
**Inspection of *ripTAL CRDs* indicates the molecular mechanisms shaping *ripTAL CRD* composition.** Closely related repeats are colored in the same color, with exception of gray that does not indicate any relatedness. **(A)** Specificity altering SNPs. Repeats 10-13 of RipTALI-1 and RipTALI-8 are shown. The RVDs and cognate codons of repeat 12 of each RipTAL are further given in yellow boxes. The two depicted SNPs constitute the only polymorphisms between these two repeats. **(B)** Repeat duplication by segmental gene conversion. Repeats 7–11 of RipTALI-9 are shown, as well as repeats 7-12 of RipTALI-1 and RipTALIII-1. Green color indicates HD repeats that are highly similar in sequence, within each array. A less related HD repeat is displayed in dark gray. Proposed segmental gene conversion events are indicated by dashed lines with arrowheads. Polymorphic bases between *repeats 8, 10, and 11* of the respective *ripTAL* are displayed to the right of the cartoon display. Next to the base comparison, repeat RVD, as well as the position within the array, colored according to the fill color of that repeat in the cartoon display is given. **(C)** Duplication of adjacent *repeats* by slipped-strand mispairing exemplified on *ripTALII-1*. Repeats 1-6 of a proposed ancestral repeat array are shown to the left. A slipped-strand mispairing DNA intermediate is shown above. Repeats 1–7 of the resulting product are shown to the right. Slipped-strand mispairing leads to a duplication of the repeat colored in orange. **(D)** A recombination event leads to loss of all repeats except one in *ripTALI-7*. The remaining repeat is fusion of *ripTALI-1* repeats 1 (pink) and 16 (blue). To the right, polymorphic bases of *repeats 1* and *16* are shown. The *ripTAL* designation, repeat RVD and repeat position are given to the right of the sequence of polymorphic bases, colored according to the fill color of that repeat.

Inspection of RipTALs I-1 and I-8 reveals that these differ only in repeat 12 by an SH (I-1) to NP (I-8) RVD polymorphism (**Figure 2**). The corresponding 105 bases of *repeat 12* from *ripTALI-1* and *ripTALI-8* differ by only two substitution polymorphisms present in their RVD codons [position 12 (AGC→AAC, translating to S→N], position 13 [CAT→CCT, translating to H→P)] (**Figures 2, 4, 8A**, and **9A**). Since RVDs define DNA binding specificity the observed RVD changes will possibly cause differences in affinity to certain DNA sequences and therefore impact on the activation of different host genes. This might explain why most RVDs are conserved within RipTAL CRDs from strains with similar host-ranges.

#### Insertion of Individual Repeats within an Array

The *ripTALIII-1 CRD* contains four HD repeats (**Figure 2**). HD repeats 10 and 11 are neighbors, which seems to suggest that these evolved by duplication. However, inspection of *ripTALIII-1 repeats* 10 and 11 shows that these differ in 11 out of 105 bases (corresponds to 90% homology). By contrast *repeats 10* and *8* of *ripTALIII-1* are sequence identical. We thus assume that *repeat 10* evolved via segmental gene conversion from *repeat 8* or reciprocally that *repeat 8* evolved via segmental gene conversion from *repeat 10* (**Figure 9B**).

RipTALI-1 shows, like RipTALIII-1, three HD repeats in positions 8, 10, and 11 and one might assume that its *CRD* evolved in the same way as the *ripTALIII-1 CRD*. However, in *ripTALI-1 repeat 8* is only 92% identical to *repeat 10* but 97% identical to *repeat 11* (**Figure 7**). This suggests that in *ripTALI-1 repeat 8* evolved by segmental gene conversion of *repeat 11*, or vice versa (**Figure 9B**).

The CRD of RipTALI-9 is similar to the RipTALI-1 CRD but displays HD repeats only in positions 8 and 10 but not in position 11 (**Figure 2**). Therefore, we consider it likely that *repeat 8* of *ripTALI-1* is the ancestor of *repeat 11*.

The differences in *repeat* identities observed for *ripTALI-8* and *ripTALIII-1* are indicative of independent, convergent evolution of the *CRDs* to yield two highly similar *repeat arrays*, by duplication of the same ancestral *repeat* (*repeat 8*) into two distinct positions *(10* or *11*), giving rise to DNA binding domains, which confer binding of highly similar, if not identical sequences (**Figures 2, 4B, 5, 8**, and **9**) in independently evolving broad host-range strains. In sum our data suggest that the identical RVD composition that we observed in two distinct RipTALs (I-8 and III-1) originating from strains in two geographically separated habitats are the consequence of convergent evolution.

#### An Elevated GC Content in the TALE, but Not RipTAL CRDs, Suggests Frequent Gene Conversion in TALEs

Frequent gene conversion is known to increase GC content in the affected region, known as GC-biased gene conversion (Lassalle et al., 2015).

A comparison of the GC contents of *TALE* and *ripTAL* across the complete CDS shows 66–67% GC content for both (Supplementary Table S3). However, the GC content of *TALEs* is not homogenous across the CDS. The *TALE NTR* and *CTR* (65 and 61% GC, respectively) are GC poor compared to the *CRD* (70%). By contrast, *ripTAL*s show less fluctuation in GC content across the *CDS* (Supplementary Table S3), indicative of less frequent gene conversion acting on the *CRD*, relative to *TALEs*. In sum, the elevated GC contents in *TALE CRDs* as compared to *ripTAL CRDs* suggests that gene conversion occurs more frequently in *TALEs* as compared to *ripTALs*.

#### Duplication of Repeats by Slipped-Strand Mispairing

We find evidence for direct *repeat* duplication when comparing *repeats 4* and *5* of *ripTALII-1* (**Figure 8B**; Supplementary Figure S5). These *repeats* are 100% identical, indicative of a recent duplication event (**Figure 8B**; Supplementary Figure S5). Similarly, *repeats 3* and *4* of *ripTALIV-2* as well as *repeats 2* and *5* of *ripTALIV-2* are 100% identical and are thus also likely the result of duplication events (Supplementary Figures S4b and S5). *Repeats 2/5* and *3/4* of *ripTALIV-2* differ only by a single nucleotide. It thus seems possible that all four *repeats* were generated from a single progenitor by multiple slipped-strand mispairing events.

#### Loss of the CRD

*ripTALI-7* consists of one *repeat* only and is likely the product of *repeat* loss. We conclude this based on alignment of *repeats 1* and *16* from *ripTALI-1* to the single *ripTALI-7 repeat* that indicate that this single repeat appears to be a fusion of the first and last *repeats* of for example *ripTALI-1* (**Figure 9C**) with a concordant deletion of all intervening *repeats*. This may have occurred via strand slippage on the template strand, leading to looping out and loss of *repeats* 1–15, or it may have occurred via intra-molecular recombination.

## Discussion

### RipTALs Appear to be an Ancestral Feature of the Rssc but Are Not Part of the Core Effector Depertoire

Our study of the natural diversity and abundance of *ripTALs* within the Rssc (**Figure 1A**) uncovered that *ripTALs* are present in all phylotypes, although abundance differs between the four phylotypes. *ripTALs* appear to be prevalent in phylotypes I and IV (**Figure 1A**) and are rare in phylotypes II and III. The absence of *ripTALs* from most but not all phylotype II and III strains suggests that the gene is ancestral and has been lost in multiple lineages. It has been suggested that *ripTAL*s were horizontally transferred into the Rssc, possibly from a *Xanthomonas* strain, and subsequently transferred horizontally to certain other lineages within the species complex (Fall et al., 2007; Heuer et al., 2007). However, the pairwise similarities we observe for RipTAL NTR and CTR sequences of different phylotypes (**Figure 1C**) show the same relationships as the species complex phylotypes (Wicker et al., 2012), and also reflect the individual taxonomic species. This observation is consistent with a model where a *ripTAL* progenitor was present in the last common ancestor of the species complex.

### The Ability of Some RipTALs to Activate the Same Sequence Evolved Convergently

We showed that the similarities of predicted RipTAL EBEs lead to cross-activation of corresponding promoters by different RipTALs (**Figure 5**). For example, RipTALI-1, RipTALI-8, and RipTALIII-1 (**Figure 5**), all coming from strains infecting a broad range of solanaceous host plants activate promoters containing any of the corresponding EBEs. As illustrated in **Figure 9**, separate events likely gave rise to the DNA binding domains of RipTALI-1, RipTALI-8, and RipTALIII-1, suggesting that these RipTALs have evolved convergently toward the same target sequence. This may imply that not only the RipTAL EBEs but also the downstream-encoded disease-promoting host proteins are conserved across many solanaceous host species.

Unlike the generalist strains found across the Rssc, some strains from phylotypes II and IV are highly host-specific and are epidemiologically restricted to *Musa* species (Ailloud et al., 2015, 2016), causing Moko and blood disease of banana, respectively. Moko disease is prevalent in Latin America and has been reported to cause yield losses of up to 100% (Munar-Vivas et al., 2010). Blood disease is mainly found in Indonesia, where it has been reported in 90% of all provinces (Hadiwiyono et al., 2007) and causes yield losses up to 100% (Supriadi, 2005). Both diseases are the result of host-specialization yet must have evolved in distinct, geographically separated phylogenetic groups.

RipTALIV-2 was isolated from all BDB strains we analyzed and RipTALII-1 was isolated from the Moko disease causing strain Molk2 (**Figure 2**; Supplementary Table S1). Recent genome sequencing of additional Moko disease causing strains indicates that *ripTALIIs* are also present in some of these strains (Ailloud et al., 2015). Given that abundance of *ripTALs* is otherwise low in phylotype II strains, the presence of ripTALs in multiple Moko disease causing strains might suggest that these RipTALs make a disease contribution in banana.

In our initial comparison of RVDs (**Figure 2**) we aligned all RipTALs starting with the N-terminal NT repeat. Based on this alignment we did not expect overlap in promoter activation for RipTALII-1 (Moko disease) and RipTALIV-2 (blood disease). We observed, however, that both RipTALs are able to significantly activate the EBE_IV-2 containing promoter (**Figure 5**) and tried to rationalize this observation. We noted that RipTALII-1 fits almost perfectly to EBE_IV-2 if binding occurs two bases downstream from the predicted G_0_ for RipTALIV-2 (Supplementary Figure S6). In sum, the unexpected cross-activity of RipTALII-1 and RipTALIV-2 may suggest a common host target gene in banana.

### RipTAL Convergence May Provide an Opportunity to Generate Disease Resistant Plants

It seems conceivable that RipTALs, like TALEs, promote virulence via host *S* gene activation. Thus mutations in corresponding RipTAL EBEs will disrupt RipTAL-mediated activation and eliminate the disease-promoting function of the RipTAL.

Given that RipTALs from strains causing Moko and blood disease may target the same EBE, in a yet to be identified banana *S* gene, mutations in this EBE might mediate protection against both, Moko and blood disease. Similarly, RipTALs from broad host range strains of phylotypes I and III appear to activate the same promoters. Identification of the disease relevant target gene and abolishing the transcriptional up-regulation of said target by modification of the EBEs, may render solanaceous hosts more resistant to RipTAL bearing strains of phylotypes I and III. Notably, mutations of EBEs in TALE targeted rice *S* genes resulted in rice cultivars resistant to *Xanthomonas oryzae* pv. *oryzae* (Li et al., 2012) suggesting that the same strategy could be applied to host-adapted Rssc strains that rely on RipTALs during infection.

### TALE-Likes of Plant Pathogens in an Ecological Context and Implications for TALE-Based Resistance Engineering

When comparing RipTALs to TALEs, it is important to consider the differences in lifestyle of the pathogens. Depending on the overall host range of a pathogen and the availability of hosts, different ecological models apply. For example, if a given RipTAL of a broad host range population is recognized in a certain host genotype, the affected population might persist by colonizing a nearby alternative host. Thereby, retention of a RipTAL that promotes disease in many, but triggers immunity in one of many hosts, may be advantageous for a broad host-range pathogen, which also has many non-crop weed hosts. Based on these considerations there will be selective pressure to maintain the binding specificity of RipTALs. However, at the population level mutations in *ripTALs* may also cause diversification into strains with different host specificities. We envision that mutations in *ripTALs* that otherwise trigger plant defense in a certain host could facilitate colonization of these otherwise Rssc resistant hosts. This would allow colonization of one host while impairing the ability to grow in others within the previous, broader range. For example, all sequenced ginger-adapted phylotype I strains carry the non-functional RipTALI-7, which only contains a single repeat, in their genome^3^, which possibly indicates that presence of a functional RipTAL might be detrimental in this specific host. More generally this observation may suggest that functional changes in a RipTAL correlate to host-adaptation (**Figure 5**), an hypothesis that was previously formulated based on a correlation between repeat number in the CRD of RipTALs and strain host-range (Heuer et al., 2007).

TALEs found in xanthomonads often differ in RVD composition, and thus target sequence, but the individual repeats are near identical otherwise (Supplementary Figure S4). In a recent report, Booher et al. (2015) showed that within closely related rice infecting *Xanthomonas* strains *TALE* content and genomic position of *TALE*s are similar, but that the diversity of their CRDs is high, and that their *CRDs* evolve quickly. CRD polymorphisms are restricted largely to number of repeats and RVD type. Similar observations were made by Pérez-Quintero et al. (2015) who used RVD compositions as the basis to construct phylogenetic trees for *TALE*s and compared these to *NTR* and *CTR* based trees. The comparisons showed that trees constructed from RVD sequences, used as proxy for CRDs, are different from those constructed based on NTR and CTR, again implying differential evolutionary behavior of the individual regions. Collectively, these observations indicate that the *TALE CRD* evolves faster than the *NTR* and *CTR*, pointing toward a selective pressure promoting evolvability of the *CRD*. This is similar to the *vlsE* locus of Lyme disease *Borrelia*, that confers antigenic variability in these pathogens. In *vlsE*, a central region of elevated variability and increased GC content is embedded in a flanking, invariable region (Norris, 2015; Supplementary Figure S3).

The TALE CRD itself acts as a direct interface between pathogen and host, and the sequence specificity of the CRD can be rapidly altered by alterations of the CRD specific RVD sequence, while retaining the sequence and structure of the remaining repeat residues. Graves et al. (2013) have described a similar situation for the *vlsE* locus, driving antigenic variation in the bacterial pathogen *Borrelia*, where natural selection promotes an antigenic variation system exhibiting a high evolvability while retaining structure. By promoting diversity in residues exposed to the host, while constraining diversification of the structural scaffold, the molecular host-pathogen interface remains highly flexible (Graves et al., 2013). This is conceptually similar to the TALE CRD, where diverse RVDs are embedded in conserved non-RVD scaffolds (Boch and Bonas, 2010).

In summary, it appears that the TALE CRD and antigenic variation systems, as employed by *Borrelia*, make use of the decreased genetic stability, and consequent increased evolvability, of repetitive sequences. However, these conserved repetitive sequences are precisely interspersed with variable sequences.

During molecular interaction with the host, the variable sequences define the outcome of the interaction. Shuﬄing and exchange of these variable epitopes embedded in conserved structural scaffolds can give rise to new interaction outcomes and enable the adaptation to new host genotypes, or to the changing antibody complement in one host.

Why then do we find reduced evolvability, due to low repeat-identity, in *ripTALs*? Strains of the *R. solanacearum* species complex are often able to infect multiple plant families and this is likely to cause different selection pressure as opposed to narrow host range pathogens as for example *Xanthomonas*.

While the near-identical repeats of *Xanthomonas* TALEs may allow for fast adaptation, there is a trade-off as their highly repetitive sequence makes them inherently unstable genes. *ripTALs*, with their lower inter repeat-sequence identities may be less prone to recombination. This is possibly an adaptive advantage if there is little variation in the, so far unknown, RipTAL target gene promoter across multiple host plants. Based on our finding that RipTALs with overlapping DNA-targeting specificities are found in strains adapted to different solanaceous hosts (**Figure 5**; Supplementary Table S1), we hypothesize that a RipTAL EBE in a given host target gene is conserved across multiple solanaceous host plants. Should this hypothesis hold true, generation of more tolerant or even resistant crop plants by alterations in the yet to be identified host EBE, could be used to generate bacterial wilt resistance in a number of different host plants

## Author Contributions

NS, OdL, and TL designed the experiments. PP provided material and NS and OdL performed the experiments. NS, OdL, and TL analyzed the data and all authors interpreted the data. NS wrote the manuscript with input from all authors.

## Conflict of Interest Statement

The authors declare that the research was conducted in the absence of any commercial or financial relationships that could be construed as a potential conflict of interest.

## Footnotes

1 https://iant.toulouse.inra.fr/bacteria/annotation/site/prj/T3Ev2/

2 www.r-project.org

3 https://iant.toulouse.inra.fr/bacteria/annotation/site/prj/T3Ev2/, https://www.genoscope.cns.fr/agc/microscope/home/index.php, strains SD54 and SD58 (de Lange et al., 2013)
