# Supplementary figures and images for: TALE-Like Effectors Are an Ancestral Feature of the Ralstonia solanacearum Species Complex and Converge in DNA Targeting Specificity
Source: Front Plant Sci. 2016 Aug 17;7:1225. doi: 10.3389/fpls.2016.01225 (PMC4987410; doi:10.3389/fpls.2016.01225)

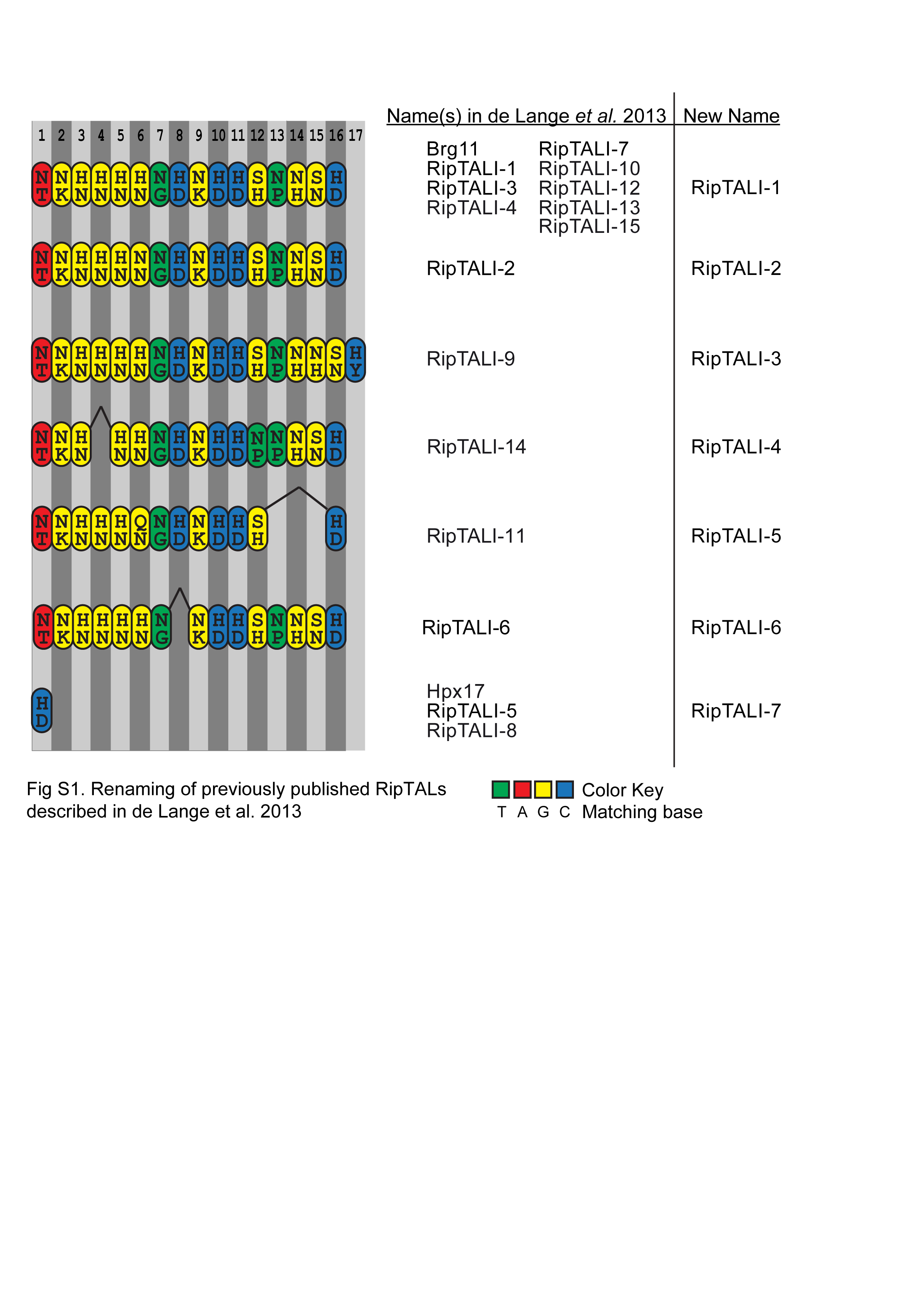

Supplement: Supplementary file 4 [file Image_1.TIF]

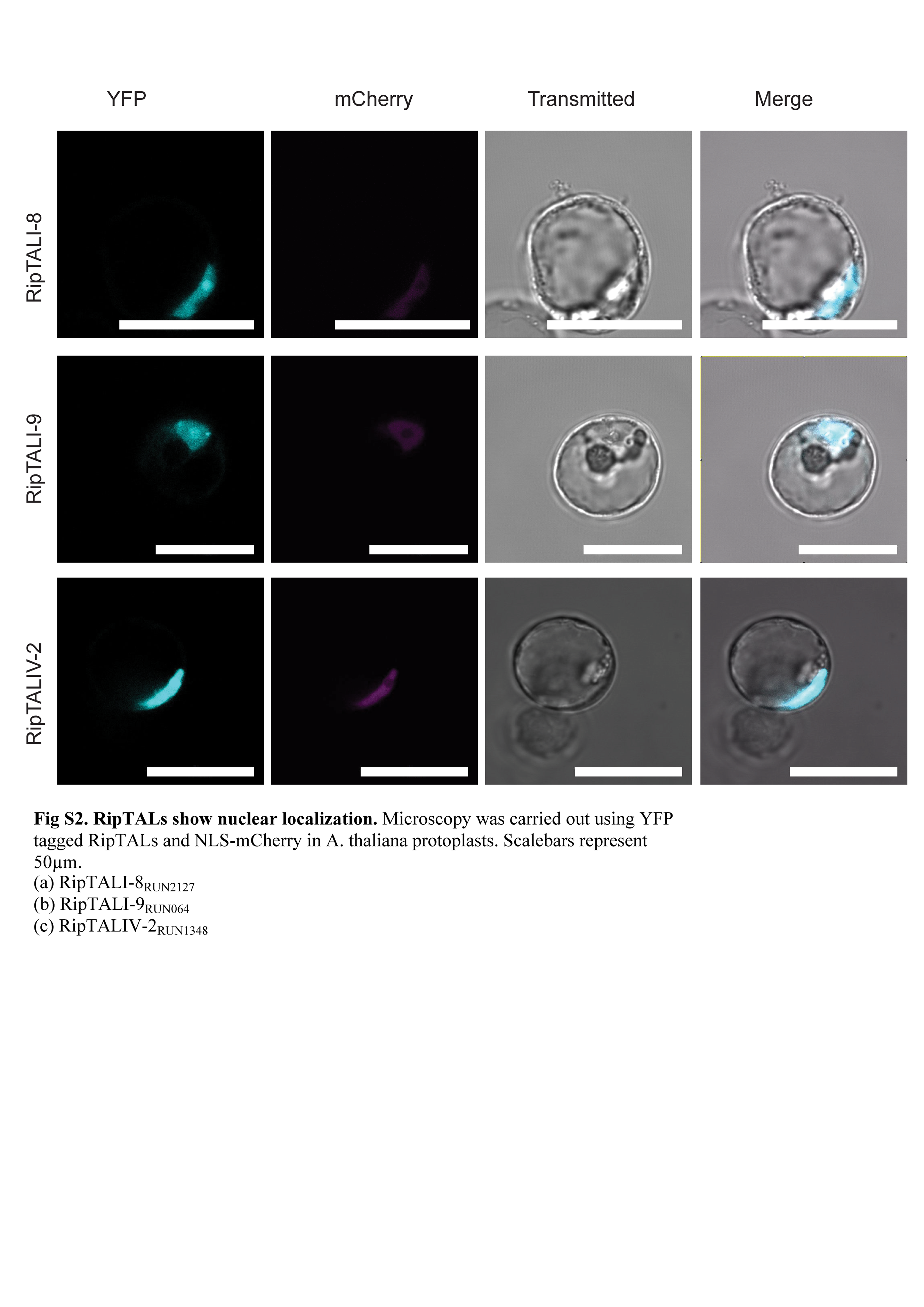

Supplement: Supplementary file 5 [file Image_2.TIF]

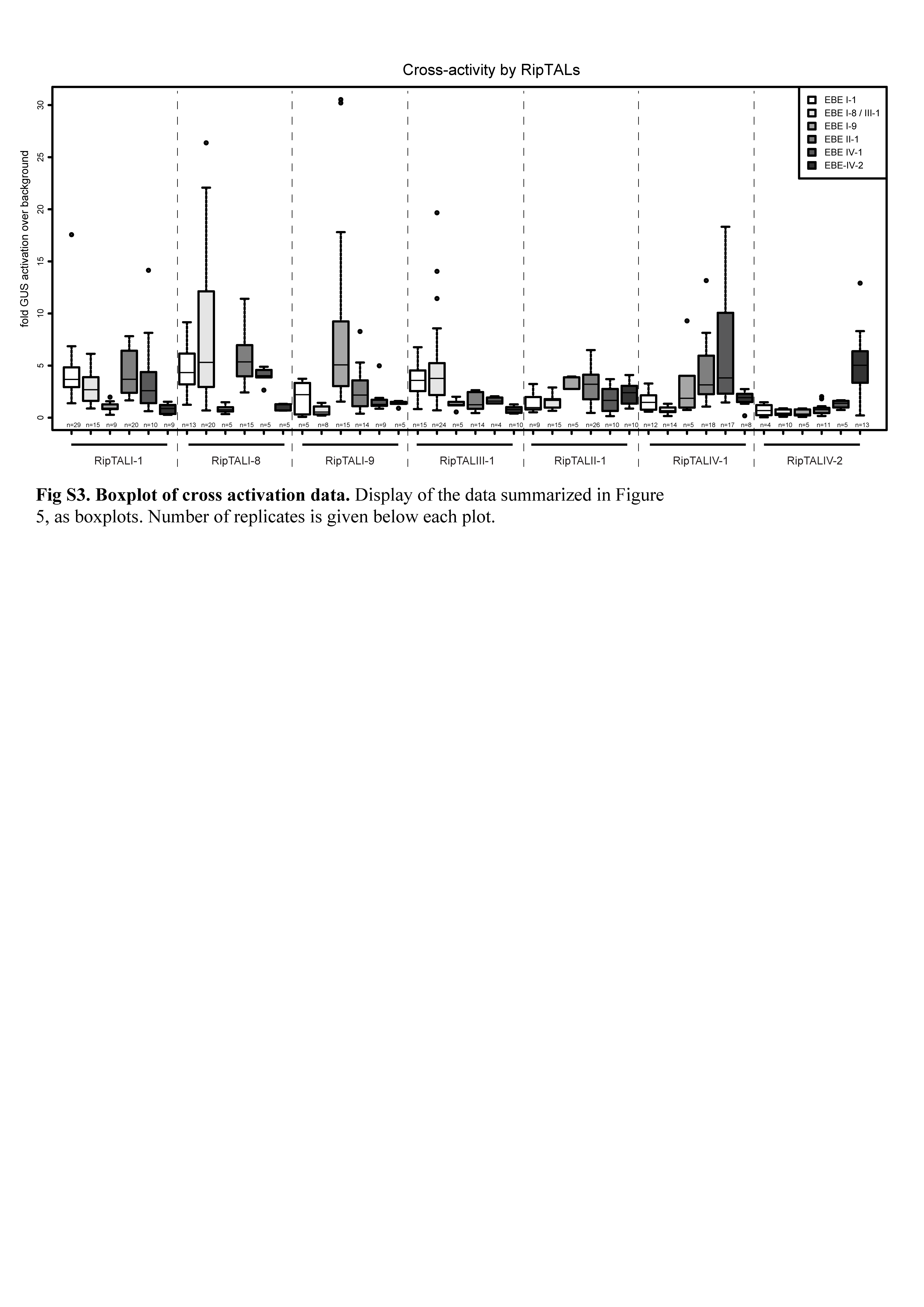

Supplement: Supplementary file 6 [file Image_3.TIF]

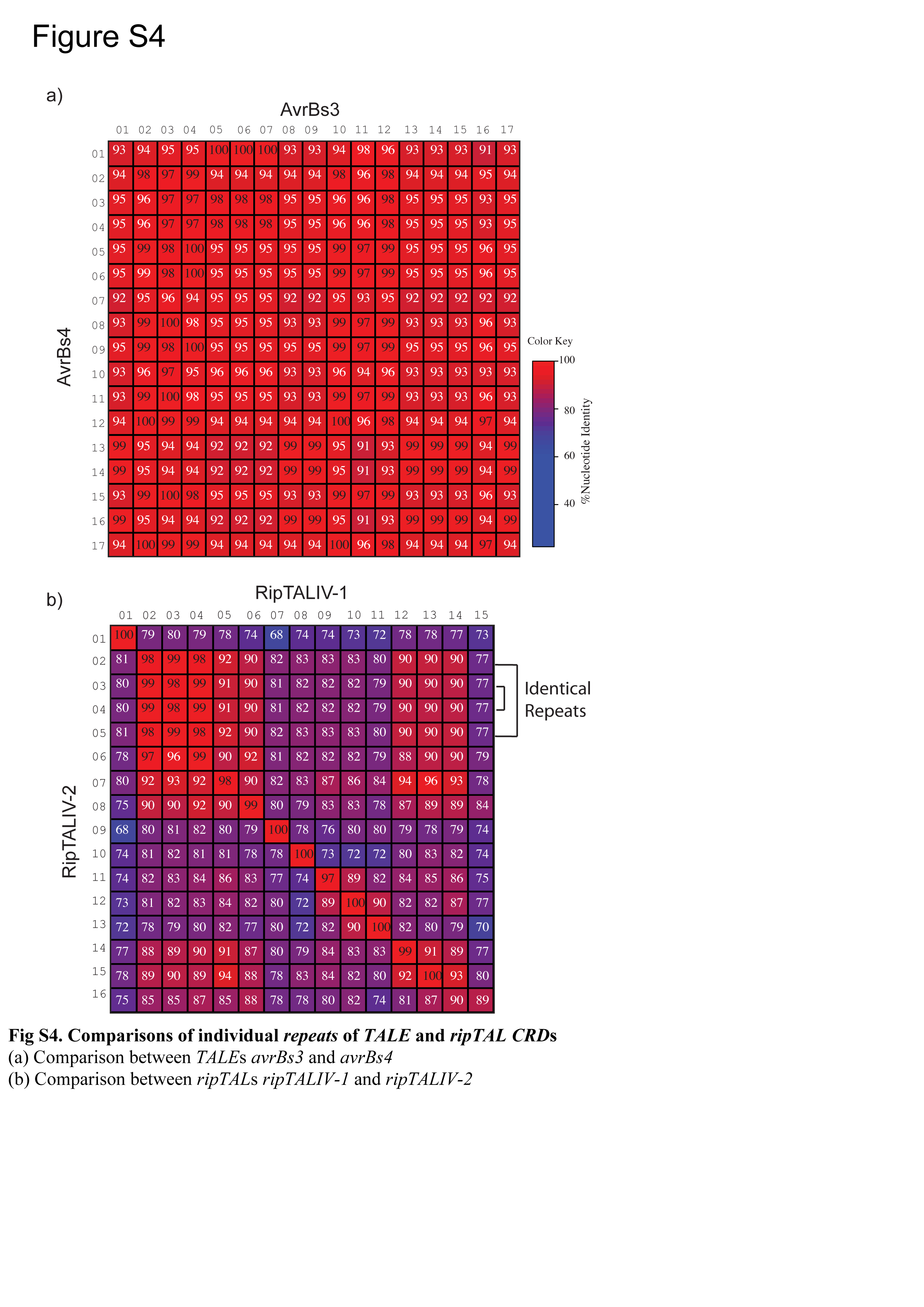

Supplement: Supplementary file 7 [file Image_4.TIF]

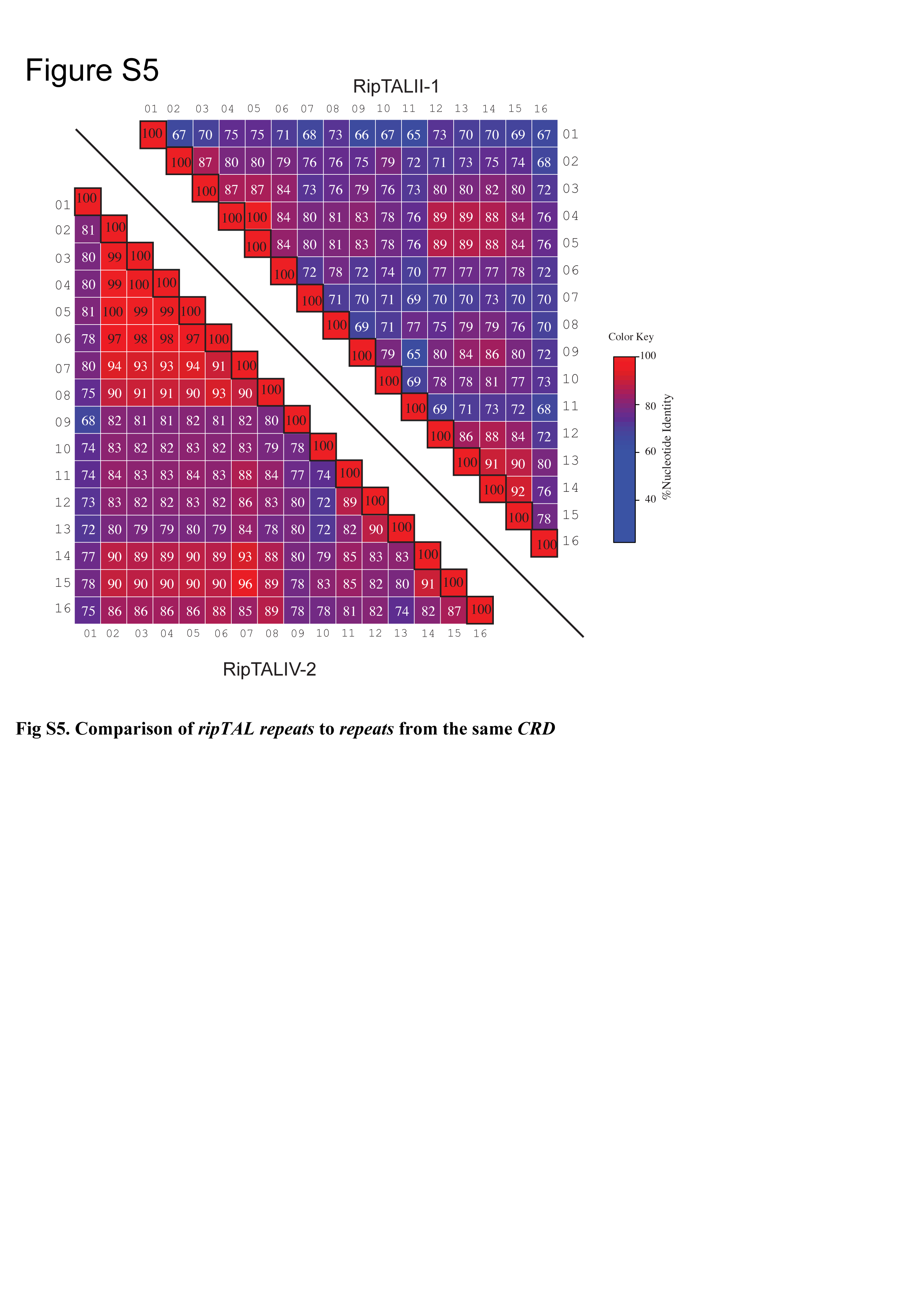

Supplement: Supplementary file 8 [file Image_5.TIF]

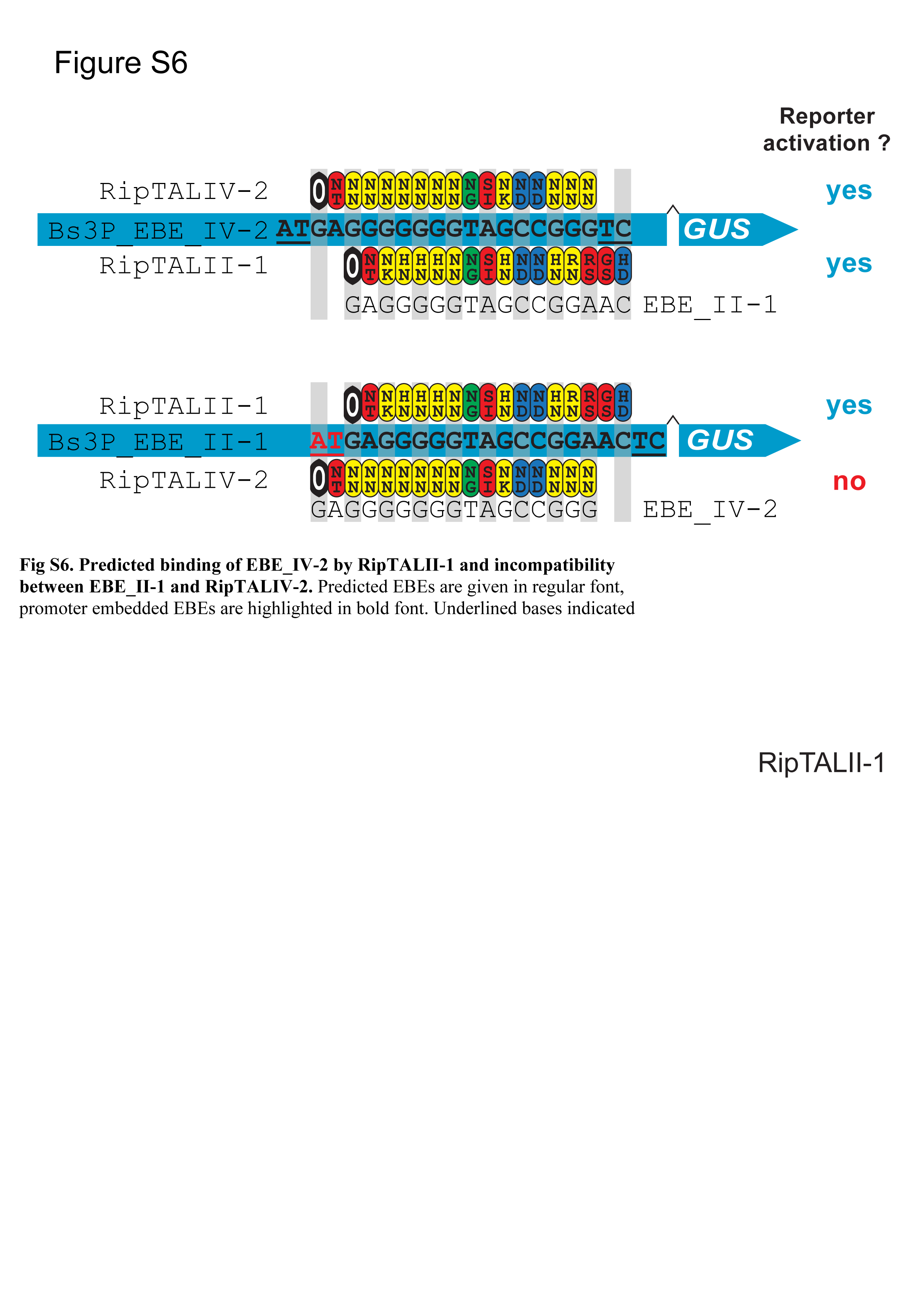

Supplement: Supplementary file 9 [file Image_6.TIF]
